# Assessment of Energy and Nutrient Intake and the Intestinal Microbiome (ErNst Study): Protocol and Methods of a Cross-sectional Human Observational Study

**DOI:** 10.2196/42529

**Published:** 2023-04-07

**Authors:** Andreas Dötsch, Benedikt Merz, Sandrine Louis, Carolin Krems, Maria Herrmann, Claudia Dörr, Bernhard Watzl, Achim Bub, Andrea Straßburg, Ann Katrin Engelbert

**Affiliations:** 1 Department of Physiology and Biochemistry of Nutrition Max Rubner-Institut-Federal Research Institute of Nutrition and Food Karlsruhe Germany; 2 Department of Nutritional Behaviour Max Rubner-Institut-Federal Research Institute of Nutrition and Food Karlsruhe Germany

**Keywords:** dietary assessment, human observational study, nutrient intake, human intestinal microbiome, biomarkers, 24-hour recall, validity, nutrition, diet, assessment, food, behavior

## Abstract

**Background:**

On the national level, nutritional monitoring requires the assessment of reliable representative dietary intake data. To achieve this, standardized tools need to be developed, validated, and kept up-to-date with recent developments in food products and the nutritional behavior of the population. Recently, the human intestinal microbiome has been identified as an essential mediator between nutrition and host health. Despite growing interest in this connection, only a few associations between the microbiome, nutrition, and health have been clearly established. Available studies paint an inconsistent picture, partly due to a lack of standardization.

**Objective:**

First, we aim to verify if food consumption, as well as energy and nutrient intake of the German population, can be recorded validly by means of the dietary recall software GloboDiet, which will be applied in the German National Nutrition Monitoring. Second, we aim to obtain high-quality data using standard methods on the microbiome, combined with dietary intake data and additional fecal sample material, and to also assess the functional activity of the microbiome by measuring microbial metabolites.

**Methods:**

Healthy female and male participants aged between 18 and 79 years were recruited. Anthropometric measurements included body height and weight, BMI, and bioelectrical impedance analysis. For validation of the GloboDiet software, current food consumption was assessed with a 24-hour recall. Nitrogen and potassium concentrations were measured from 24-hour urine collections to enable comparison with the intake of protein and potassium estimated by the GloboDiet software. Physical activity was measured over at least 24 hours using a wearable accelerometer to validate the estimated energy intake. Stool samples were collected in duplicate for a single time point and used for DNA isolation and subsequent amplification and sequencing of the 16S rRNA gene to determine microbiome composition. For the identification of associations between nutrition and the microbiome, the habitual diet was determined using a food frequency questionnaire covering 30 days.

**Results:**

In total, 117 participants met the inclusion criteria. The study population was equally distributed between the sexes and 3 age groups (18-39, 40-59, and 60-79 years). Stool samples accompanying habitual diet data (30-day food frequency questionnaire) are available for 106 participants. Current diet data and 24-hour urine samples for the validation of GloboDiet are available for 109 participants, of which 82 cases also include physical activity data.

**Conclusions:**

We completed the recruitment and sample collection of the ErNst study with a high degree of standardization. Samples and data will be used to validate the GloboDiet software for the German National Nutrition Monitoring and to compare microbiome composition and nutritional patterns.

**Trial Registration:**

German Register of Clinical Studies DRKS00015216; https://drks.de/search/de/trial/DRKS00015216

**International Registered Report Identifier (IRRID):**

DERR1-10.2196/42529

## Introduction

Within the last decade, there has been a huge increase in research in the field of the human microbiome, leading to a new body of knowledge in this area. It is now well established that the human intestinal microbiome is an essential mediator in the interplay between diet and host physiology. Initiatives like the HDHL-INTIMIC Knowledge Platform on Food, Diet, Intestinal Microbiomics, and Human Health [1] were founded to collect and extend this knowledge by fostering multidisciplinary projects.

Many associations between diet and the microbiome have been described, for example, correlations of the long-term diet with differences in gut microbiota composition and functions [2-7]. However, until now, only a small part of gut microbiota variation has been attributed to nutrition [8]. The usual dietary pattern has a strong influence on microbiota composition, as has been shown for plant-rich diets such as the Mediterranean diet, vegetarian or vegan habits, and the abundance of *Prevotella* species [9-12]. It is important to understand how nutrition impacts microbiota, as a disturbance in the latter (dysbiosis) has been associated with many diseases, including diet-related diseases such as obesity and type 2 diabetes. Many studies have been performed on such specific topics, but the results are often inconsistent or even contradictory [13-15]. Such a variety of results can be partially explained by differences in the study population, but also by a lack of standardization of methods, including sample preparation, technology, and bioinformatic analysis [16-18].

Besides the importance of standard methods to determine the microbiome composition, the assessment of reliable representative dietary intake data also requires standardized and validated tools that are up-to-date with recent developments in food products and the nutritional behavior of the population. In Germany, the Max Rubner-Institut (MRI) is responsible for the national nutrition monitoring of the adult population and has so far conducted the second German National Nutrition Survey [19] and the longitudinal study NEMONIT [20]. National nutrition monitoring serves to assess food consumption, nutritional status, and nutritional behavior of the general population and specific population groups. In addition, trends in the nutritional behavior of the population can be monitored. The data are used to update national food-based dietary guidelines, to perform risk assessment by food consumption of the population toward chemical substances in food items, and to serve as decision support for national and European statutory rules. Sound and representative data on nutrition are, therefore, essential for policy-making, answering scientific questions, providing information on the population, and making national and international comparisons.

In this context, the European Food Safety Authority (EFSA) developed methodological guidelines for data assessment in national food consumption surveys [21,22]. Accordingly, 24-hour recalls should be applied to national nutrition monitoring as a dietary assessment method for the adult population. The software GloboDiet (previously EPIC-Soft; International Agency for Research on Cancer), developed as a software platform for a standardized, structured interview, fulfills the EFSA recommendations and thereby provides data with the required detail and quality for national nutrition monitoring [23,24]. In some countries across the European Union, GloboDiet is used for the assessment of representative detailed food consumption data [25,26]. GloboDiet was adapted for the German food market. For example, new foods available on the food market were added and obsolete foods were deleted. In addition, adjustments were made to the description and quantification of the foods in accordance with the abovementioned objectives of the German National Nutrition Monitoring. The standard units to assess the consumed quantity of different food items were updated, and the descriptions of the foods were adjusted to enable analyses of the consumption of ultraprocessed foods.

The ErNst (“Erfassung der Energie- und Nährstoffzufuhr”; English “assessment of energy- and nutrient intake”) study was conceived to address two independent objectives: (1) to verify whether food consumption, as well as energy and nutrient intake of the German population, can be recorded validly by means of the current version of the dietary recall software GloboDiet for the German National Nutrition Monitoring and (2) to obtain biosamples and high-quality data for an exploratory assessment of associations between dietary intake, microbiome composition, and microbial metabolites (as a proxy for functional activity of the microbiome). The purpose of this manuscript is to provide a comprehensive description of the study protocol and the methods used for the collection of biosamples and dietary intake data, combined with an overview of the number of samples and data points that will be available for further analysis.

## Methods

### Study Design and Setting

**Figure 1:**
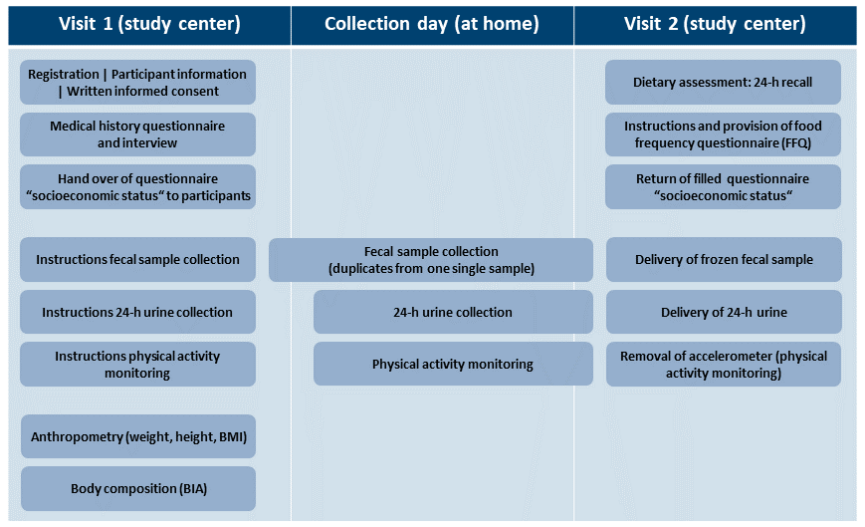
Overview of the ErNst (Erfassung der Energie- und Nährstoffzufuhr) study days. Collection day was either on the first or second day after visit 1. Visit 2 followed immediately the day after collection day. BIA: bioelectrical impedance analysis.

The ErNst study was designed as a cross-sectional observational study and performed at the Division of Human Studies of the Department of Physiology and Biochemistry of Nutrition at the MRI, Karlsruhe, Germany, between October and December 2018. On day 1, all volunteers visited the study center for registration, further instructions, application of the accelerometer device, and initial measurements (visit 1, Figure 1). Furthermore, participants were informed about the study procedures and gave their written consent (see also Ethics Approval). Either on the first or second day after the initial visit, participants collected stool and 24-hour urine samples at home (collection day) and brought them on the second visit to the study center the day after (visit 2). Questionnaires and interviews on nutritional intake were also completed on this day.

During visits 1 and 2, the participants underwent a series of standardized examinations described in detail below. For all interviews, examinations, measurements, sample collection, sample preparation, and storage, standard operating procedures were developed and tested for feasibility. Interviewers and examiners had been extensively trained and supervised during data collection.

### Participants

Female and male volunteers aged 18-79 years without any known or apparent acute or chronic illnesses or diseases were eligible to participate. Detailed inclusion and exclusion criteria are listed in Textbox 1. Health status questionnaires were used to assess medical history, including potential confounding variables like the use of antibiotics within the last 12 months and smoking status.

**Figure 2:**
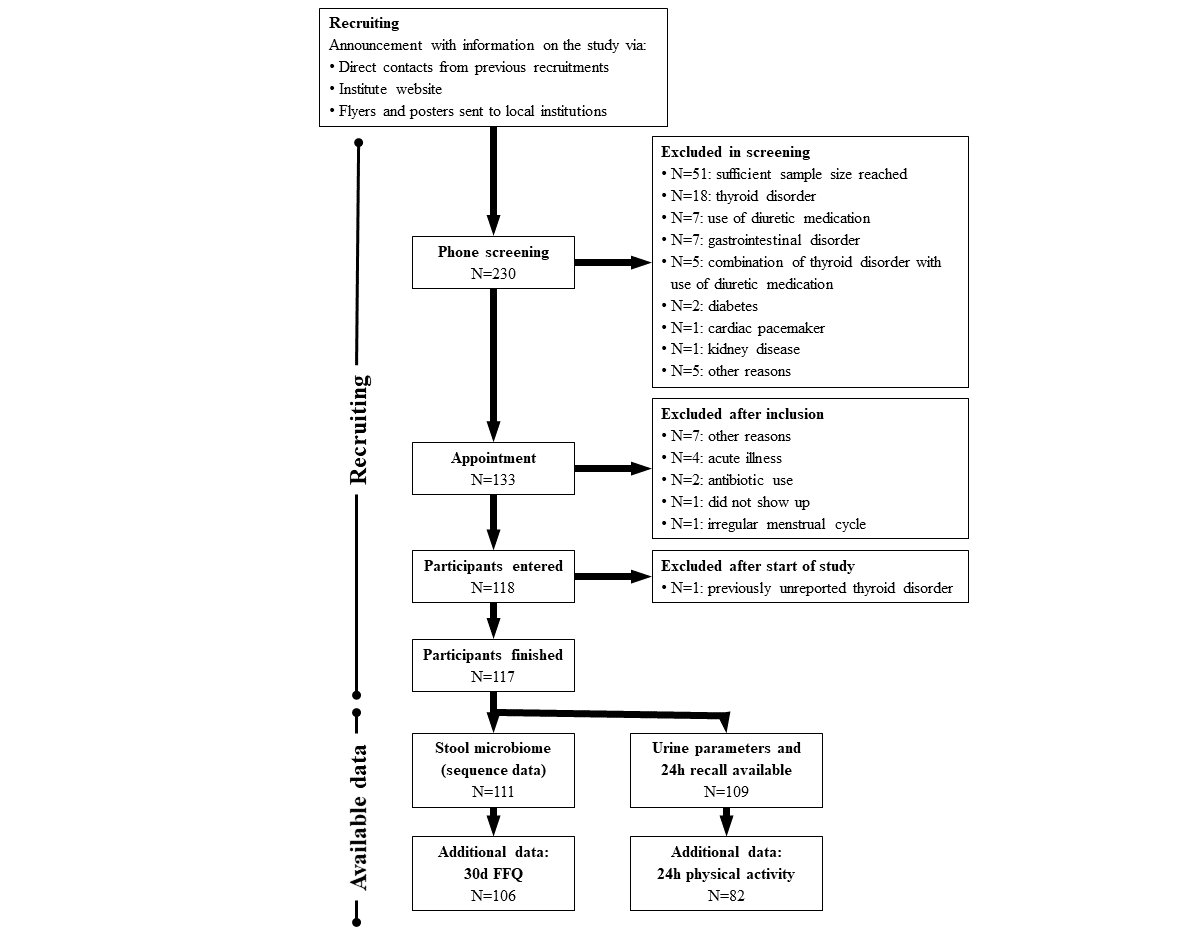
Flowchart describing the recruitment process and available data points of the ErNst (Erfassung der Energie- und Nährstoffzufuhr) study. The bifurcation of the available data separates long-term and short-term (current) data points. FFQ: food frequency questionnaire.

Recruitment procedures included direct communication with previous study participants and MRI employees and their families, the use of flyers and other information materials provided to institutions in and around Karlsruhe, information about the study on MRI’s homepage, and word of mouth. Initially, 230 interested persons contacted the study center (Figure 2). Those who passed an initial telephone interview (done with a checklist) were scheduled for their first study visit (n=133). Reasons for the exclusion of interested persons are also given in Figure 2. Participants included in the study were asked to complete a questionnaire regarding their marital status, partner relationship status, household size (the number of people living in the household, including children), net monthly household income, school education (highest school-leaving qualiﬁcation), and occupational qualification.

Since the software GloboDiet shall be validated for the German National Nutrition Monitoring of the adult population, we recruited the participants of the ErNst study according to 3 preselected age groups (18-39, 40-59, and 60-79 years). The proportion of men and women in the respective age groups was equal, as was the total number of participants in each of the 3 groups.

**Criteria for inclusion and exclusion.**

**Inclusion criteria**

- Healthy female or male
- Able to complete all planned examinations, interviews, and questionnaires
- Written consent to participate voluntarily in the study
- Aged between 18 and 79 years

**Exclusion criteria**

- Pregnant or breastfeeding
- Diabetes mellitus
- Renal disease
- Use of diuretics
- Hyper- or hypothyreosis
- Currently on diet to reduce weight
- Technical implants (such as pacemaker)
- Amputation of arms or legs
- Diseases and treatments leading to malabsorption of energy and nutrients
- Blood donation within last 4 weeks
- Insufficient German language to complete interviews
- Use of antibiotics within last 12 months
- Tumors requiring therapy
- Acute or chronic infectious disease
- Abuse of drugs or alcohol
- Likely failure to comply with study conditions
- Persons who are kept in an institution by court or official order
- Failure or unwillingness to provide written consent of participation

### Anthropometric Measurements and Assessment of Body Composition

Anthropometric data were assessed using standard methods. Participants were asked not to eat or drink for 4-5 hours before the examination.

Body weight was measured to the nearest 0.05 kg in light clothing without shoes, and height was measured to the nearest 0.1 cm using the seca 285 measurement station (seca). Both parameters were used to calculate the respective BMI (kg/m²).

Bioelectrical impedance analysis (BIA) was used to assess body composition with the measurement device Nutriguard-MS (Data Input GmbH) using multifrequency technology (5.50-100 kHz; 0.8 mA). The analysis was conducted with NutriPlus software (version 5.3.0; Data Input GmbH).

### Assessment of Physical Activity and Calculation of Total Energy Expenditure

The activity energy expenditure (AEE) was determined using accelerometers (ActiHeart from CamNtech) for at least 24 hours. Here, the heart rate was recorded together with the acceleration data, so that activities that took place in a sitting position and where the subject itself experienced little acceleration (eg, cycling) could also be adequately depicted. The software used for programming and reading out the acquired data was the ActiHeart software (version 4.0.131; CamNtech).

Resting metabolic rate (RMR) was calculated according to the formula of Müller et al [27] and considered fat-free mass as well as fat mass and the age of the participants.

Dietary-induced thermogenesis (DIT) accounts for about 10% of the energy content of the consumed food [28-30]. Total energy expenditure (TEE) was calculated using the following formula: TEE (kcal) = RMR (kcal) + AEE (kcal) + DIT (kcal)

### Assessment of Current Food Consumption by Applying the Software GloboDiet (24-Hour Recall) and Calculation of Energy and Nutrient Intake

During the second visit to the study center, the food consumption of the participants was assessed face-to-face by trained interviewers using the software GloboDiet. The software was adapted to the current German food market and modified for use in the German National Nutrition Monitoring of the adult population. The structure and standardization procedures of GloboDiet have been described in detail elsewhere [24]. For the 24-hour recall, participants were asked in detail about their food and beverage consumption, as well as the use of dietary supplements on the previous day. Corresponding to a first so-called quick list of the consumed foods and beverages in chronological order of the meals, the reported food items were further described by facets and descriptors (eg, the applied preparation method, the preservation method, the flavor, and the fat content). Consumed quantities were assessed with a picture book, household measurements, as well as standard units. Interviews were conducted on Mondays, Wednesdays, and Thursdays, leading to results of food consumption on Sundays, Tuesdays, and Wednesdays, representing a balanced coverage of weekdays and weekends, which was intended regarding the validation of the GloboDiet software.

Based on the assessed food consumption, energy and nutrient intake were calculated using data from the German Nutrient Database version 3.02 [31].

### Acquisition of Habitual Diet (30-Day Food Frequency Questionnaire)

The 48-item food frequency questionnaire (FFQ) that was used in this study is an adapted version of the validated 53-item FFQ applied in the German Health Examination Survey for Adults 2008-2011 [32]. The ErNst FFQ, therefore, includes questions on the frequency and amount of food groups consumed during the past 4 weeks. The questionnaire was given and explained to the participants on their second visit to the study center, with the request to complete it at home and return it. Frequencies for the consumption of food items were asked according to prespecified categories: never, once a month, 2-3 times a month, 1-2 times a week, 3-4 times a week, 5-6 times a week, 1 time per day, 2 times per day, 3 times per day, 4-5 times per day, and more than 5 times per day. Furthermore, participants had to report the portion sizes of the food items consumed in predefined answering categories. Portion sizes were based on common household measures such as a cup, a glass, a tablespoon, and so on, and, where applicable or necessary, supported by pictures to aid participants in estimating their usual intake. Habitual nutrient intake was calculated using the German Nutrient Database version 3.02 [31].

### 24-Hour Urine Collection

The 24-hour collection of urine by the study participants took place the day before the 24-hour dietary recall (visit 2). The study participants were provided with 2 urine collection containers (volume 2 liters, Sarstedt AG & Co) and were asked to store the urine at home in the refrigerator or in a cool bag with prechilled thermal packs to keep the collected urine permanently cool. This cool bag was also used to ensure the chilled transport of the urine collection until delivery to the study center. Urine collection was performed according to a standard protocol. On the first visit to the study center, participants were instructed by a study nurse and were given a protocol with written instructions and documentation. Subjects were interviewed about any difficulties during 24-hour urine collection on the second visit day (delivery of 24-hour urine). Urine samples were excluded from the analysis in case of collection errors, for example, if the collection time was interrupted, incomplete, or less than 20 hours, or if the first-morning urine was not discarded as described in the standard protocol.

Creatinine was analyzed as a reference value for potential urinary analytes using a method modified from Slot [33] and Heinegård and Tiderström [33,34] with the Urinary Creatinine Detection Kit from Arbor Assays (Ann Arbor) according to the manufacturer’s protocol. The creatinine excretion in urine was also used to estimate the completeness of the urine collection. Creatinine is formed during the breakdown of creatine or creatine phosphate. This occurs almost exclusively in muscle tissue; therefore creatinine excretion in the urine is related to muscle mass or fat-free body mass. Since creatinine excretion in urine is subject to intraindividual fluctuations and depends on the creatine or creatinine content of food [35], it is only suitable for a rough estimate of the completeness of the 24-hour urine collection.

### Stool Sampling and Storage

Study participants were provided with a prepacked stool collection kit (“Easy Sampler,” GP Medical Devices A/S) and received precise oral instructions and a written manual for the sampling procedure. Each participant collected 2 independent, approximately nut-sized samples from the same stool. Samples were frozen at –20 °C immediately after collection, delivered frozen to the study center, and then stored at –80 °C until aliquoting. After all the samples were collected, multiple aliquots of 200 mg and 500 mg were kept at –80 °C until further analysis. One aliquot per sample was immediately transferred to and frozen in NucleoSpin Bead Tubes Type A (Macherey-Nagel GmbH & Co) for DNA extraction (see Microbiome Analysis).

### Analysis of Biomarkers in Urine

#### Overview

The validation of the German GloboDiet version will use an approach with nitrogen and potassium urinary excretion as biochemical markers for protein, as well as potassium intake [36,37]. Nitrogen and potassium excretion were chosen because errors are likely to be independent between the measurements of biomarkers and dietary intake [38,39].

#### Nitrogen Content

Total urinary nitrogen was quantified by the Kjeldahl method using automated digestion and distillation units (Digestion Unit K-35, KjelFlex K360, Büchi). Concentrated sulfuric acid and Hg- and Se-free Kjeldahl catalyst tablets (Merck) were used for digestion. Titration was done with 0.05 M sulfuric acid. Individual urine volumes and an assumed specific gravity of urine of 1.020 g/mL were used to convert the nitrogen content into grams of nitrogen per 24 hours. The reference range for the specific gravity of urine is between 1.010 and 1.030 g/mL. The Karlsruhe Metabolomics and Nutrition (KarMeN) cohort [40] was used as the reference population.

#### Sodium and Potassium Content

Sodium and potassium measurements were performed in an accredited clinical laboratory (MVZ Labor PD Dr Volkmann and colleagues) using ion-sensitive electrodes.

In order to obtain the excreted urinary sodium and potassium amount per day, the respective concentrations (mmol/L) were converted into g/d while taking the volume of urine and the respective molar masses into account.

#### Osmolality

Osmolality in urine was measured using the freezing point osmometer, Advanced Osmometer Modell 2020 (Advanced Instruments LLC), and values were expressed in mOsm/kg. These values can be used as reference values for potential urinary analytes to be measured in the future.

### Analysis of Stool Samples (Intestinal Microbiome)

Single aliquots of the 2 independent stool samples collected from each subject were used for DNA extraction using the NucleoSpin DNA Stool Kit (Macherey-Nagel). The 200 mg of stool, previously transferred to the bead tubes, were incubated with 850 µL lysis buffer at 70 °C for 5 minutes. A bead beating step was included using a FastPrep 24 machine (MP Biomedicals) at 6.0 m/s for 40 seconds, followed by washing and elution according to the kit’s standard protocol. The purity and concentration of the extracted DNA were checked on a NanoDrop microvolume photometric device. The variable region V4 of the prokaryotic 16S rRNA gene was amplified in a single polymerase chain reaction (PCR) step with dual indexing primers as described by Kozich et al [41] with a modification to increase sensitivity toward Archaea [42] (Multimedia Appendix 1). Phusion Hot Start II DNA Polymerase (Thermo Fischer Scientific) was used in a 25 µL reaction mix with 1 ng template DNA, 0.5 µM of each PCR primer (forward and reverse), 0.2 mM dNTP mix, 0.25 µL enzyme, and 5 µL 5x HF buffer. The PCR program included initial denaturation for 2 minutes at 98 °C, 25 cycles of 30 seconds at 98 °C, 10 seconds at 55 °C, and 15 seconds at 72 °C, followed by terminal elongation for 5 minutes at 72 °C. PCR products were purified using magnetic beads (Mag-Bind RXNPure Plus, Omega) and their concentration was measured using a Quantus Fluorometer (Promega) with the QuantiFluor One dsDNA System (Promega), according to the manufacturer’s recommendation. The PCR products were pooled at an equimolar concentration in 2 independent libraries that were subsequently sequenced on the Illumina MiSeq platform using a MiSeq Reaction Kit v3 with 2 × 301 cycles at a final concentration of 8 pM, adding 20 mol% of PhiX DNA.

### Sample Size Calculation

The sample size was based on the validation of the 24-hour recalls using the difference between energy expenditure (EE) and energy intake (EI) as the end point. The statistical power for a paired *t* test of the hypotheses H_0_: EI – EE = 0, H_1_: EI – EE ≠ 0 was calculated by the Leibniz Institute for the Social Sciences (GESIS) in Mannheim, Germany. Statistical parameters were estimated using data from a study by Biltoft-Jensen et al [43], as this study used comparable methods for the estimation of EE and nutrient excretion. For an effect size *d* = (EE – EI)/SD (EE – EI), significance level α=.05, and group sizes of 50 female and 50 male participants, the statistical power was calculated to be >0.998. To prevent an age bias, the group sizes were distributed evenly to the 3 age groups, that is, recruiting at least 17 participants per sex and age group.

The second aim of the study, the analysis of microbiome composition and its association with nutrition, is exploratory and not hypothesis-driven. Therefore, no additional power or sample size calculations were required for this part of the study.

### Ethics Approval

The study has been performed in accordance with the Declaration of Helsinki. It was approved by the Ethics Committee of the State Medical Chamber of Baden-Württemberg (EK LÄK BW; F-2018-055) in October 2018 and registered at the German Clinical Trials Register (DRKS00015216). All participants were informed in detail about the purpose of the study, examination procedures, measurements, and potential risks involved. Participants signed their consent to the procedures and the analysis of pseudonymized data, including their potential use for research related to nutrition and health. Privacy and confidentiality of personal data are guaranteed by following the regulations of the State Medical Chamber and the EU General Data Protection Regulation. Pseudonymization was achieved by assigning randomized subject identifiers. All participants received €50 (US $56.50) in compensation upon inclusion in the study.

## Results

Recruitment of potential participants involved an initial telephone screening of 230 people who were interested in participating, of whom 133 were selected based on exclusion criteria (Figure 2). After their assignment to the study, 16 subjects had to be excluded for various reasons (Figure 2). Overall, a total of 117 participants completed the 2 visits to the study center (Table 1, Multimedia Appendix 2).

For 109 participants (57 women and 52 men), food consumption and nutrient intake data, assessed by a face-to-face 24-hour dietary recall, are available. The participants were equally distributed among the age groups (18-39 years of age: 36; 40-59 years of age: 35; 60-79 years of age: 38). Urine samples collected during a 24-hour period were provided and suited for analysis in 109 cases (nitrogen and osmolality measurements failed in 2 cases that could not be validly reproduced).

ActiHeart records were not complete in 27 cases, mostly because of the detachment of the device or similar technical problems that resulted in measurements shorter than the required 24 hours. Physical activity data were obtained for 82 of the 109 participants for which urine and current food consumption data were collected.

Nearly all participants provided the requested stool samples, but a few reported irregularities during sampling (eg, potential contamination with toilet water) that led to the exclusion of the samples from further analyses. A final available number of 111 stool samples, each consisting of 2 replicates, were attained.

FFQs were completed by 106 participants (53 women and 53 men) who also provided valid stool samples, equally distributed over the age groups (18-39 years of age: n=37; 40-59 years of age: n=31; and 60-79 years of age: n=38).

**Table 1:** Study population characteristics (N=117).

Characteristics		Female participants, n (%)^a^	Male participants, n (%)^a^
**Age group (years)**			
	18-39	21 (17.9)	18 (15.4)
	40-59	19 (16.2)	19 (16.2)
	60-79	21 (17.9)	19 (16.2)
**Smoking**			
	Never	55 (47.0)	52 (44.4)
	Occasionally	4 (3.4)	2 (1.7)
	Daily	2 (1.7)	2 (1.7)
**BMI**			
	<25.0	43 (36.8)	29 (24.8)
	25.0-29.9	12 (10.3)	24 (20.5)
	≥30.0	6 (5.1)	3 (2.6)
**School education^b^**			
	≤9 years	5 (4.3)	4 (3.4)
	10-11 years	13 (11.1)	7 (6.0)
	≥12 years	43 (36.8)	45 (38.5)

^a^Of whole study population.

^b^Highest school-leaving qualiﬁcation, recoded to years spent in school.

## Discussion

### Principal Findings

Within the study, 117 participants completed the recruitment. After the subtraction of some errors in sample collection and measurements, 106 stool samples are available with corresponding data on common nutrition (30-day FFQ). In addition, 109 face-to-face 24-hour recalls were conducted (data on current nutrition), with corresponding urine samples and measured urine parameters. For 82 of these data sets, additional data on physical activity have been collected.

The 2 main aims of the study were to collect samples and data for (1) validating the dietary recall software GloboDiet and (2) for studying the association between diet and the intestinal microbiome. The 117 participants who successfully completed the study were equally distributed with respect to sex and age groups. However, the fraction of participants with a BMI above 25 kg/m² was lower in the study (females: 18/61, 29.5%, males: 27/56, 48.2%) than observed in the official representative statistics of the German population in 2017 (females: 43.1%, males: 62.1%, data set 12211-9018 in the Genesis database [44]). In addition, the average level of education is high in the study population. Therefore, the study population could be somewhat biased toward a healthier lifestyle, including nutrition and activity, than would be expected in the general population.

For the validation of the updated GloboDiet version, protein and potassium intake determined with the software will be compared with the excretion of protein and potassium in the 24-hour urine collection. In addition, EI calculated from the 24-hour recall will be compared to TEE and RMR. To estimate the validity of the current GloboDiet version, different methods will be applied, like the Wilcoxon-signed rank test to test if the differences between nutrient excretion and nutrient intake, as well as TEE and EI, are different from zero, Spearman rank correlation coefficients to assess the strength of the correlations, as well as Bland-Altman plots [45,46]. Bland-Altman diagrams are suitable for visualizing measurement differences. In addition, participants will be grouped into tertiles to assess the agreement in ranking participants regarding nutrient intake and excretion, as well as EI and TEE. Furthermore, for EI, cutoff values according to Goldberg et al [47] and Black [48] will be calculated for group levels as well as for individuals.

With the samples collected in this study, we plan to further analyze the association between habitual diet and gut microbiota. The microbiota analysis will include single parameters (alpha-diversity and abundance of single taxa), which will be compared with specific food groups or nutrient intakes to identify biologically significant associations. More complex data, like beta diversity of the microbiome and dietary patterns, will be compared by means of ordinations or group categories like enterotype and dietary pattern (using both a priori and a posteriori approaches, such as principal component and principal coordinate analysis, reduced rank regression, or dietary indices). Thereby, we also aim at identifying which level of dietary data yields potential explanations for the observed microbiota variations (eg, dietary pattern, food group, or nutrient level). As a similar composition of the gut microbiota can lead to different activities and functions of this complex and versatile ecosystem within the host, it is important to look not only at the present bacteria but also at the metabolites they produce. Therefore, the fecal content of short-chain fatty acids and bile acids will also be determined. Correlation analyses between nutrition parameters and these functional indicators of microbial activity will further enhance the obtained information on associations between the microbiome and nutrition.

Besides the 2 primary aims of the study, the set of valuable samples and data offers opportunities for further analyses. The availability of 24-hour urine samples with corresponding information on nutrient intake, for example, enables the search for biomarkers associated with specific foods or food constituents. This data can be further enhanced or validated using data from the KarMeN study that was previously performed at the MRI [40].

### Limitations

The study population was not recruited representatively. Therefore, the average BMI of study participants was lower than generally observed in the German population, indicating a potential bias toward healthier nutrition and lifestyles among the participants. With regard to recruitment, the focus was based on the sample size of the study population needed for the validation of the 24-hour recalls. A total of 50 women and 50 men provided sufficient statistical power for the planned analyses. However, the number of participants was comparatively low for the analysis of the microbiome, limiting the resolution to identify fewer common associations, for example, between nutrition and microbial features. Microbiome analysis based on 16S-rRNA genes is limited to identifying taxonomic composition, but available samples offer opportunities to measure metabolite concentrations or sequence the metagenomes to enable deeper insights.

A further limitation is that TEE was not measured using a doubly labeled water technique. This method has been shown to provide an accurate measure of the TEE [49-51]. At the time when the study was conducted, the use of doubly labeled water was not possible due to time and cost constraints.

### Comparison With Prior Work

During the KarMeN study, the metabolomes of blood and urine samples from 301 participants were analyzed, enabling the identification of biomarkers for dietary intake [40]. In comparison, while lacking blood samples, ErNst includes urine samples that are available for metabolomics and adds the opportunity to measure the intestinal microbiome and metabolome using the collected stool samples.

### Conclusions

We completed the recruitment and collection of samples and data for the ErNst study with a high degree of standardization. This enables the validation of the GloboDiet software, which will be used in the German National Nutrition Monitoring. In addition, microbiome composition and nutritional patterns will be compared to investigate functional associations and potential biomarkers.

## Appendix

Multimedia Appendix 1 List of primers used for amplification and sequencing of the V4 region of the prokaryotic 16S-rRNA gene.

Multimedia Appendix 2 Overview of availability of samples and data points per study participant.

## Abbreviations

AEE: activity energy expenditure
DIT: dietary induced thermogenesis
EE: energy expenditure
EFSA: European Food Safety Authority
EI: energy intake
ErNst: Erfassung der Energie- und Nährstoffzufuhr
FFQ: food frequency questionnaire
KarMeN: Karlsruhe Metabolomics and Nutrition
MRI: Max Rubner-Institut
PCR: polymerase chain reaction
RMR: resting metabolic rate
TEE: total energy expenditure

## Data Availability

In accordance with the informed consent of the ErNst (Erfassung der Energie- und Nährstoffzufuhr) participants, pseudonymized study data could be provided to external researchers upon request for cooperation or data-use purposes. In this case, a cooperation or data-use agreement must be concluded in advance. The use of ErNst study data is therefore possible with a detailed prior justification of its scientific purpose and after agreement with the MRI. However, informed consent would, in general, allow the publication of a publicly available data set of anonymized study data, which is also planned in the future.

Raw sequence data have already been deposited in the European Nucleotide Archive [52] under the accession number PRJEB51875. The study was included in the collection of human observational studies in the context of the INTIMIC Knowledge Platform [53], and study metadata were deposited on the project’s metadata server, Mica [54]. Further data will be published later with the results of the ongoing analyses and could be shared earlier upon a reasonable request.
